# Synanthropic Mammals as Potential Hosts of Tick-Borne Pathogens in Panama

**DOI:** 10.1371/journal.pone.0169047

**Published:** 2017-01-06

**Authors:** Sergio E. Bermúdez, Nicole Gottdenker, Aparna Krishnvajhala, Amy Fox, Hannah K. Wilder, Kadir González, Diorene Smith, Marielena López, Milixa Perea, Chystrie Rigg, Santiago Montilla, José E. Calzada, Azael Saldaña, Carlos M. Caballero, Job E. Lopez

**Affiliations:** 1 Departamento de Investigación en Entomología Médica, Instituto Conmemorativo Gorgas de Estudios de la Salud, Ciudad de Panamá, Panamá; 2 Department of Veterinary Pathology, College of Veterinary Medicine, University of Georgia, Athens, Georgia, United States of America; 3 Department of Pediatrics, Section of Tropical Medicine, Baylor College of Medicine and Texas Children’s Hospital, Houston, Texas, United States of America; 4 Departamento de Investigación en Parasitología, Instituto Conmemorativo Gorgas de Estudios de la Salud, Ciudad de Panamá, Panamá; 5 Parque Municipal Summit, Ciudad de Panamá, Panamá; 6 El Níspero, El Valle, Coclé, Panamá; University of Kentucky College of Medicine, UNITED STATES

## Abstract

Synanthropic wild mammals can be important hosts for many vector-borne zoonotic pathogens. The aim of this study was determine the exposure of synanthropic mammals to two types of tick-borne pathogens in Panama, spotted fever group *Rickettsia* (SFGR) and *Borrelia* relapsing fever (RF) spirochetes. One hundred and thirty-one wild mammals were evaluated, including two gray foxes, two crab-eating foxes (from zoos), four coyotes, 62 opossum and 63 spiny rats captured close to rural towns. To evaluate exposure to SFGR, serum samples from the animals were tested by indirect immunofluorescence assay (IFA) using *Rickettsia rickettsii* and *Candidatus* Rickettsia amblyommii antigen. Immunoblotting was performed using *Borrelia turicatae* protein lysates and rGlpQ, to assess infection caused by RF spirochetes. One coyote (25%) and 27 (43%) opossums showed seroreactivity to SFGR. Of these opossums, 11 were seroreactive to *C*. R. amblyommii. Serological reactivity was not detected to *B*. *turicatae* in mammal samples. These findings may reflect a potential role of both mammals in the ecology of tick-borne pathogens in Panama.

## Introduction

Synanthropic mammals are a diverse group of wild animals that prosper in areas where humans are present, both in rural and urban conditions [1]. Rodents, opossum, and mid-sized wild carnivores inhabit or migrate throughout ecotones that contain forest, pasture, and human dwellings, and may be important components of the transmission ecology of different pathogenic microorganisms [2, 3]. Furthermore, blood-feeding ectoparasites such as ticks add a dimension of complexity to the ecology of infectious diseases. In North America and Europe, there is evidence of tick-borne pathogens (TBP) are associated with wild mammals close to anthropogenic areas [4, 5, 6]. Unfortunately, throughout Central America there is a paucity of information regarding the roles of synanthropic mammals in the transmission of TBP.

There is evidence regarding the prevalence and significance of TBP in Panama [7, 8]. The first findings of TBP were cases of relapsing fever (RF) *Borrelia* in the early 1900s [9], with more than 100 cases confirmed in Panama City and neighboring localities [10]. Moreover, detection of RF spirochetes in the blood of small and large mammals including opossums, monkeys, armadillos, horses, and calves was also reported in the country [11, 12]. Currently, it remains unclear whether the pathogens continue to circulate in Panama.

From 1950–1953 five confirmed cases of *Rickettsia rickettsii* spotted fever were reported in Panama [13, 14, 15], while eight were diagnosed between 2004–2014 [8, 16, 17, 18]. Of these 13 cases, nine were fatal. In addition, there are also records of the *Candidatus* “Rickettsia amblyommii,” a member of the spotted fever group *Rickettsai* (SFGR) and other TBP such as ehrlichiosis and anaplasmosis in domestic mammals and ixodid ticks [18, 19, 20].

Given that TBP pose a risk to human public health in Panama, it is important to understand the role of mammals as putative hosts. In this current study, we evaluated serological responses of three carnivore species (coyotes, grey foxes, crab-eating foxes), common opossums, and spiny rats to SFGR and RF *Borrelia* antigens in Panama. Our findings indicate the importance of further defining the ecology of TBP in Panama.

## Materials and Methods

### Field sites

From 2013–2015, samples were taken opportunistically from wild carnivores relocated from anthropogenic localities to zoos: four coyotes (*Canis latrans*), two gray foxes (*Urocyon cinereoargenteus*) in El Níspero zoo (Coclé province), and two crab-eating foxes (*Cerdocyon thous*) in Summit Municipal Park (Panamá province). One coyote (No. 4) was removed from a highway two days prior to sampling. The animal was weak and lethargic, presumably hit by a car. However, traumatic injuries were not found; yet a skin rash was observed in trunk, abdomen, and inguinal area. Table 1 indicates origin of coyotes and foxes analyzed in the study. After chemical immobilization [intramuscular acepromazine (0.5 ml), plus ketamine 10% (0.5 ml) for coyotes], all animals were evaluated for the presence of ticks and additional ectoparasites. Blood was drawn from the femoral vein of each wild canid.

**Table 1:** Sites where wild canids were originally captured.

Species	Zoo location	Time in zoo^a^	Capture site	Characteristic	Ticks
*Canis latrans*	El Níspero	>300	Natá, Coclé	Rural town	No
*Canis latrans*	El Níspero	>300	Natá, Coclé	Rural town	No
*Canis latrans*	El Níspero	>180	Chepo, Panamá	Rural town	No
*Canis latrans*	El Níspero	2	Arraiján, Panamá Oeste	Urban town around secondary forest	Yes ^b^
*Cerdocyon thous*	Summit MP	Unknown	Unknown	Unknown	No
*Cerdocyon thous*	Summit MP	Unknown	Unknown	Unknown	No
*Urocyon cinereoargenteus*	El Níspero	~30	Santiago, Veraguas	Urban city, around pasture areas	No
*Urocyon cenereoargenteus*	El Níspero	~ 30	Santiago, Veraguas	Urban city, around pasture areas	No

^a^ Days that each animal was in the zoo prior to the extraction of blood.

^b^ Ticks collected were 101 *Amblyomma* cf. *oblongoguttatum* and one *Rhipicephalus sanguineus* s.l.

**Fig 1:**
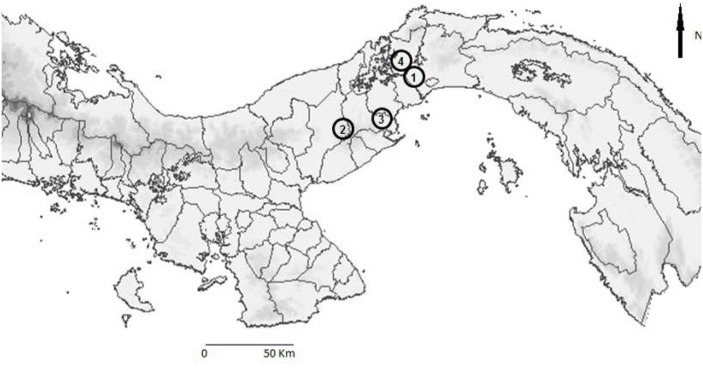
Map of Panama showing the geographical sites: Summit Municipal Park (1), El Níspero (2), Capira (3), Gamboa (4).

In addition, during 2013–2014, Tomahawk and Sherman traps were used to catch small mammals in the towns Gamboa (9° 07´25´´ N– 79° 43´ 02´´ W) (Colon province), Trinidad de las Minas (8° 46′ 32″ N—79° 59′ 45″ W), and Las Pavas (9° 6’ 15” N—79° 53’ 9” W) (Capira, Western Panama province). For the trapping and handling of the captured mammals, we followed methods cited by González et al. [21]. The ecology of Gamboa consists of rainforest [22], while Trinidad de las Minas and Las Pavas are rural towns surrounded by secondary forest and pasture [23, 24]. Fig 1 indicates the localities. The blood samples were centrifuged and serum was collected to detect the presence of circulating antibodies to SFGR and RF *Borrelia*.

### Immunofluorescence assay (IFA) to verify previous exposure to SFGR

Serum samples were tested using crude antigens derived from Brazilian strains of *R*. *rickettsii* (Taiacu) and *C*. *“R*. *amblyommii”* (AC37) from the collection of the Center for Research in Tropical Diseases, Faculty of Microbiology at the University of Costa Rica, as previously described [25, 26]. Slides were incubated with fluorescein isothiocyanate labeled sheep anti-dog IgG, sheep anti-opossum IgG (derived from *Didelphis aurita*) (CCZ, SP, Brazil), and rabbit anti-guinea pig IgG, for wild canids, marsupials and rodents, respectively, following procedures described by Horta et al. [25] and Labruna et al. [26]. Initial screenings were at a 1 to 64 dilution, and the slides were read using an ultraviolet microscope (Nikkon Eclipse E-400) at 1,000 X magnification. Fluorescence was compared to positive and negative controls as previously described (25), and reactive samples were subsequently tested in serial two-fold dilution to determine the endpoint titer. Given the serological cross reactivity observed against species of SFGR, serum that showed a *Rickettsia* species titer of at least 4-fold higher compared to the other species was considered as the possible causative agent [26, 27]. For IFAs, negative and positive controls were from uninfected animals and laboratory infected dogs, respectively, and were provided by the University of San Palo.

### Immunoblotting to detect exposure to RF spirochetes

Protein lysates from the 91E135 strain of *B*. *turicatae* were used for SDS-PAGE and immunoblotting [28] because the strain originated from Texas and would be the closest known genetic match to species and isolates distributed in Panama. Assays were performed as previously described with 1 x 10^7^ spirochetes and 1 μg of rGlpQ electrophoresed per lane [28, 29]. All serum samples were diluted 1:200 for assays. Protein G-HRP (Life Technologies, Carlsbad, CA) or anti-opossum HRP (Alpha Diagnostics International Inc., San Antonio, TX) at a 1:4,000 dilution were used to determine serum reactivity to *Borrelia* antigens. Positive control serum samples originated from rodents and a canine infected with *B*. *turicatae* by tick bite. Animals were considered positive if serological reactivity was detected to five or more proteins in *B*. *turicatae* lysates and rGlpQ, as previously described [30].

### Ethical approval

This work was authorized by the National Bioethics Committee of Investigation (561/CNBI/ICGES/06) and Institutional Animal Care and Use Committe (IACUC, 2006/02) of the Gorgas Research Institute. Wild canids from the zoo were analyzed with authorization from the respective administrations.

## Results

Serum samples from 131 wild mammals, corresponding to the eight wild canids, 63 spiny rats (*Proechymys semispinosus*) and 62 common opossums (*Didelphis marsupialis*) were collected. Of the four coyotes, only No. 4 was reactive against SFGR; however, because this canid had similar titers for *R*. *rickettsii* and *C*. “R. amblyommii”, we could not discriminate the infectious agent (Table 2). One male of *Rhipicephalus sanguineus* s.l. and 101 *Amblyomma* cf. *oblongoguttatum* were collected from coyote No. 4, while ticks were not identified on the remaining canids (Table 1).

**Table 2:** IFA reactivity of coyote and opossum serum samples to SFGR.

No. Animal	Sites	Antigens slides		Putative pathogen ^c^
		*R*. *rickettsii* ^a^	*R*. *amblyommii* ^a^	
Coyote 4	Arraiján	2048	512	SFGR
Opossum 2	Capira	512	256	SFGR
Opossum 5	Capira	- ^b^	256	*C*. R. amblyommii
Opossum 7	Capira	512	512	SFGR
Opossum 9	Capira	- ^b^	64	SFGR
Opossum 10	Capira	- ^b^	512	*C*. R. amblyommii
Opossum 11	Capira	128	512	SFGR
Opossum 14	Capira	- ^b^	256	*C*. R. amblyommii
Opossum 18	Capira	128	1024	*C*. R. amblyommii
Opossum 19	Capira	64	64	SFGR
Opossum 22	Capira	128	1024	*C*. R. amblyommii
Opossum 27	Capira	64	128	SFGR
Opossum 28	Capira	64	64	SFGR
Opossum 33	Capira	64	1024	*C*. R. amblyommii
Opossum 35	Capira	- ^b^	64	SFGR
Opossum 37	Capira	128	1024	*C*. R. amblyommii
Opossum 43	Capira	128	128	SFGR
Opossum 46	Capira	64	128	SFGR
Opossum 49	Capira	64	1024	*C*. R. amblyommii
Opossum 50	Gamboa	128	64	SFGR
Opossum 51	Gamboa	64	- ^b^	SFGR
Opossum 53	Gamboa	128	1024	*C*. R. amblyommii
Opossum 55	Gamboa	64	64	SFGR
Opossum 58	Gamboa	128	128	SFGR
Opossum 59	Gamboa	256	1024	*C*. R. amblyommii
Opossum 60	Gamboa	- ^b^	512	*C*. R. amblyommii
Opossum 61	Gamboa	64	1024	*C*. R. amblyommii
Opossum 62	Gamboa	256	256	SFGR

^a^ Titer was defnied as the inverse of the greatest serum sample dilution.

^b^ (-) indicates that the serum sample was unreactive to the antigens used in the assay.

^c^ The SGRF designation indicates that the putative species causing infection was indistinguishable in 16 animals.

Of the 62 *D*. *marsupialis*, 27 (43%, 95% CI [31, 57%]) opossums demonstrated serological reactivity against SFGR, 18 originated from Capira (36%, 95% CI [24, 52%], N = 49) and nine from Gamboa (69%, 95% CI [39, 90%], N = 13). Of these, 12 were reactive to *C*. *R*. *amblyommii*, and 15 samples were impossible to discriminate the infectious agent (Table 2). Immature *Amblyomma* spp. and adult *Ixodes luciae* were collected from 12 opossums. None of the spiny rat (N = 63) samples were considered serologically positive SFGR, and serum samples from all the animals in this study were considered seronegative against RF *Borrelia* antigens.

## Discussion

In this study, we evaluated serological responses to SFGR and RF spirochetes in mammals that have been overlooked as putative hosts in Panama. The results indicate that opossums and coyotes may be involved with the maintenance of SFGR in the mammals tested in this study. This work compliments previous findings that SFGR circulate in Capira, a western Panama province, and the implication of opossums in fatal cases of SF in this region [15, 16]. Moreover, seroreactivity of opossums in Gamboa presents a new location within the Colon province where SFGR may circulate.

Our results further suggest the relevance of *Didelphis* species as reservoir of SFGR. In Sao Paulo, Brazil 63% of the *Didelphis aurita* (N = 65) and 72% of *Didelphis albiventris* (N = 29) were naturally infected with SFGR [25], while 100% (N = 5) of *D*. *albiventris* were seropositive to SFGR from Paulicéia, Brazil [31]. Moreover, *Didelphis* species display extended periods of rickettsemia compared to rodents, and are asymptomatic during infection with *R*. *rickettsia* [32, 33]. *Didelphis virginiana* maintained *R*. *rickettsii* for three to four weeks [32], while *D*. *aurita* remained infected with this pathogen for 26 days [33]. *Didelphis aurita* is also considered an amplifying host for immature *A*. *cajennense* ticks [33]. Given the ability of these marsupials to thrive in wild, rural, and urban environments [34], they may play a significant role in the public health significance and ecology of SFGR in Panama.

The public health significance of *C*. “R. amblyommii” remains unclear; although it may also cause mild fever in humans [35, 36, 37]. Antibody titers of 12 opossums (Table 2) suggest the animals may maintain this species of SFGR. *Candidatus* “R. amblyommii.” Additionally, there is evidence that horses and dogs maintain this species of *Rickettsia* [38, 39], and the putative pathogen has also been detected in several species of ticks in the Americas [40]. Within Panama, the organism is widely distributed and found in the *Dermacentor nitens*, *Amblyomma mixtum*, *R*. *sanguineus* s.l., *Amblyomma ovale*, and *Haemaphysalis juxtakochi* [18, 19, 41]. Future work will evaluate the ticks collected in this study for microorganism detection.

Our findings comprise the first indication in Panama that coyotes may be involved with the ecology of SFGR in the country. Although determining the *Rickettsia* species that infected coyote No. 4 was not possible, high antibody titers (2,048 *R*. *rickettsii* and 516 *C*. “R. amblyommii”), the presence of a skin rash, and the animal’s physical condition indicated an active SFGR infection. Given the close relationship between coyotes and dogs, it is possible that both canids can manifest similar symptoms to pathogenic SFGR, especially *R*. *rickettsia*. These symptoms can include skin rash, petechial hemorrhage in eyes and mucosa, lethargy and paralysis [27, 42]. Alternatively, it is known that *C*. “R. amblyommii” can stimulate an immune response in both canids [39, 43, 44], but there is no evidence to support the pathogenicity of *C*. “R. amblyommii” in dogs. Although we cannot confirm that *R*. *rickettsii* infected coyote No. 4, the symptoms presented and the seroreactivity against SFGR can potentially rule out other TBP such as ehrlichiosis and anaplasmosis that affect wild and domestic canids [45, 46].

While we failed to detect exposure to RF *Borrelia* spirochetes in the coyotes and opossums evaluated, the sample size was small and additional work is needed. These vertebrates should be considered in the ecology of RF spirochetes, as Dunn and Clark reported the detection of spirochetes in the blood of 9.8% of opossums from Panama [12]. Additionally, *Borrelia* species distributed within the United States are infectious to domestic dogs [28, 47,48], suggesting that coyotes may be a likely host. Given that the *Ornithodoros* tick vector of RF *Borrelia* possesses a 10 to 20-year life history and the observed transovarial transmission of some species [49], it is likely that the pathogens continue to circulate within Panama.

Ticks and TBP in Panama are largely neglected by health care providers and the scientific community. As infectious diseases emerge and continue to threaten human health, it is important to understand how the pathogens are maintained. Our results show insight into the ecology of SFGR in Panama, and suggest the necessity to include synanthropic mammals, such as opossums and coyotes, in future field studies. Furthermore, as additional work is focused to collect *Ornithodoros* ticks, we will define and determine the distribution and disease burden of RF spirochetes in the country.
