# Correction: Synanthropic Mammals as Potential Hosts of Tick-Borne Pathogens in Panama

**DOI:** 10.1371/journal.pone.0226195

**Published:** 2019-12-02

**Authors:** Sergio E. Bermúdez, Nicole Gottdenker, Aparna Krishnavajhala, Amy Fox, Hannah K. Wilder, Kadir González, Diorene Smith, Marielena López, Milixa Perea, Chystrie Rigg, Santiago Montilla, José E. Calzada, Azael Saldaña, Carlos M. Caballero, Job E. Lopez

The third author’s name is spelled incorrectly. The correct name is: Aparna Krishnavajhala. The correct citation is: Bermúdez SE, Gottdenker N, Krishnavajhala A, Fox A, Wilder HK, González K, et al. (2017) Synanthropic Mammals as Potential Hosts of Tick-Borne Pathogens in Panama. PLoS ONE 12(1): e0169047. https://doi.org/10.1371/journal.pone.0169047
